# Immunological memory to COVID-19 vaccines in immunocompromised and immunocompetent children

**DOI:** 10.3389/fcimb.2025.1527573

**Published:** 2025-02-17

**Authors:** Constanza Russo, Adrián Otero, Macarena Uranga, Vanesa Seery, Silvina Raiden, Silvia Algieri, Norberto De Carli, Mauricio Borda, María F. Albistur, Lourdes Heinitz, María Marcó del Pont, Martina Pardini, Guillermina Budano, Laura Alvarez, Nancy Simaz, Claudia Merhar, María C. Quintana, Cecilia Garbini, Luisa Aedo Portela, Misael Salcedo Pereira, Fernando Ferrero, Jorge Geffner, Lourdes Arruvito

**Affiliations:** ^1^ Instituto de Investigaciones Biomédicas en Retrovirus y SIDA (INBIRS), Facultad de Medicina, UBA-CONICET, Buenos Aires, Argentina; ^2^ Departamento de Medicina, Hospital Universitario Austral, Buenos Aires, Argentina; ^3^ Departamento de Medicina, Hospital General de Niños Pedro de Elizalde, Buenos Aires, Argentina; ^4^ Servicio de Pediatria, Hospital Nacional Profesor Alejandro Posadas, Buenos Aires, Argentina; ^5^ Servicio de Pediatria, Clínica del Niño de Quilmes, Buenos Aires, Argentina; ^6^ Servicio de Pediatria, Hospital Pediátrico Juan Pablo II, Corrientes, Argentina

**Keywords:** children, SARS-CoV-2, variants, vaccines, antibodies, T cells

## Abstract

**Background:**

Most children in Argentina received only the initial COVID-19 vaccine series, with presumed hybrid immunity after multiple Omicron waves. However, the durability of immune memory, particularly in immunocompromised (IC) children, remains poorly studied.

**Methods:**

A cohort of IC (n=45) and healthy children (HC, n=79) was assessed between 13 to 17 months after receiving two or three doses of BBIBP-CorV and/or BNT162b2. Plasma anti-spike IgG, neutralizing activity and antigen-specific CD4+ and CD8+ T cells against Wuhan and Omicron BA.5 variants were assessed.

**Results:**

Most children remained seropositive after two vaccine doses, but compared with HC, IC exhibited lower neutralizing titers against both Wuhan and Omicron BA.5, particularly those vaccinated with BBIBP-CorV. Even after three vaccine doses, IC showed weaker neutralizing antibody response, CD8+ T cell responses and lower IFN-γ production compared with HC. Integrated analysis of neutralizing antibodies, memory CD4^+^, and CD8^+^ T cells revealed a weak immune memory among IC with an important compromise in memory CD8^+^ T cell responses.

**Conclusions:**

Immunity can last up to 17 months, but reduced effectiveness against new variants highlights the need for updated COVID-19 vaccines, especially for IC children. Additional efforts are essential to enhance vaccination coverage and protect this vulnerable population.

## 1 Introduction

Pediatric vaccination against severe acute respiratory syndrome coronavirus 2 (SARS-CoV-2) has effectively prevented Coronavirus Disease 2019 (COVID-19)-related hospitalizations (Head et al., 2024). However, our understanding of the durability of the immune memory induced by the original monovalent vaccines targeting the Wuhan-Hu-1 strain remains limited, particularly due to low and unequal booster coverage worldwide (Eccleston-Turner and Upton, 2021; Ferranna, 2024).

According to the latest data from Argentina’s Nominalized Federal Vaccination Registry, as of August 4, 2023 (prior to the introduction of bivalent vaccines), 9.6 million children (73%) had received a first vaccine dose, 8 million (61%) a second dose, 2.6 million (20%) a first booster, and only 200,000 children (2%) a second booster. Since then, booster uptake among children has remained very low, with no updated official statistics available. Thus, it can be concluded that the majority of children in Argentina have not received any booster doses.

Given the emergence of the Omicron variant (Karim and Karim, 2021), which triggered multiple waves of infection, population immunity arises from both vaccination and Omicron breakthrough infections, resulting in hybrid immunity. Although most of the population is believed to have substantial immunity against SARS-CoV-2, immunocompromised patients remain at increased risk for severe outcomes (Petrelli et al., 2022; Meyerowitz et al., 2024).

There is limited information on the immune memory response to SARS-CoV-2 in immunocompromised children (IC), particularly those who received the whole-cell inactivated vaccine (BBIBP-CorV) (Peng et al., 2023) as their primary vaccine regimen a long time ago. This study analyzed humoral and cellular responses in immunocompromised children with various medical conditions, as well as in healthy children (HC), after receiving two or three doses of BBIBP-CorV and/or the mRNA vaccine BNT162b2 up to 17 months following the final vaccine dose.

## 2 Methods

### 2.1 Ethics statement

This study adhered to the Declaration of Helsinki and received IRB approval from participating institutions (Hospital General de Niños Pedro de Elizalde #8771/23 and Hospital Universitario Austral #P22-063). Parents or legal guardians from children under 8 years provided written, informed consent. Children older than 8 years old provided written, informed consent and their parents or legal guardians also provided written, informed consent. All samples were deidentified prior to processing.

### 2.2 Study population

This observational study was conducted at the Hospital General de Niños Pedro de Elizalde, Hospital Universitario Austral, Hospital Alejandro Posadas, Hospital Pediátrico Juan Pablo II, and Clínica del Niño de Quilmes. Two cohorts of children were enrolled, all of whom had received two or three doses of monovalent anti-SARS-CoV-2 vaccines targeting the Wuhan-Hu-1 strain. Blood samples were collected between 13 to 17 months after their last vaccine dose. The first cohort included 45 IC whose immunocompromised status was determined according to CDC criteria, including: recipients of solid organ transplants under immunosuppressive therapy; patients undergoing active cancer treatment (for tumor or blood cancers); those who had received a stem cell transplant within the past two years; children on chronic immunosuppressive therapy; and individuals with moderate to severe inborn errors of immunity (https://www.cdc.gov/covid/vaccines/immunocompromised-people.html). The second cohort included 79 HC who were vaccinated with two or three doses of COVID-19 vaccines. All children suffered SARS-CoV-2 infection between 22 and 24 months prior to obtaining blood samples. None of the participants were hospitalized or experiencing any acute active infections at the time of sampling. The characteristics of both cohorts are detailed in **Table 1**. Detailed clinical data of each immunocompromised children is presented in **Supplementary Table 1**. This study followed The Strengthening the Reporting of Observational studies in Epidemiology (STROBE) guidelines.

**Table 1:** Characteristics of study cohorts.

	Children	
	Healthy (n=79)	Immunocompromised (n=45)
Age, years, median (range)	9 (4-16)	11 (5-17)
Female, n (%)	22 (28)	15 (33)
Medical history		
Transplant	-	9 (20)
Cancer	-	15 (33)
Inborn Innate Error	-	14 (31)
Autoimmunity	-	7 (16)
COVID-19 history, n (%)	79 (100)	45 (100)
Immunosuppressive medications, n (%)	-	32 (71)
Gammaglobuline, n (%)	-	8 (18)
Doses of vaccines at sampling, n (%)		
#2	43 (54)	23 (51)
#3	36 (46)	22 (49)
Vaccination regimen, n (%)		
BBIBP-CorV (2 doses)	34 (43)	14 (33)
BNT162b2 (2 doses)	9 (12)	9 (18)
BBIBP-CorV (3 doses)	1 (1)	0 (0)
BNT162b2 (3 doses)	16 (20)	13 (27)
BBIBP-CorV (2 doses) plus BNT162b2 (1 dose)	19 (24)	9 (22)
Months post second dose, median (range)	480 (289-684)	504 (264-711)
Months post third dose, median (range)	395 (296-545)	382 (290-602)

### 2.3 Blood sample processing

Approximately 0.5-1 mL of whole blood samples were obtained. After centrifugation for 10 min at 1000 rpm, plasma was separated and stored at -80°C until use. Peripheral blood mononuclear cells (PBMCs) were isolated using Ficoll-Paque gradient (Cytiva) and cryopreserved in liquid nitrogen until use.

### 2.4 Cells and virus

VERO C1008 (clone E6, ATCC, RRID: CVCL_0574) cells were used as described (Seery et al., 2022). Wuhan variant (GISAID ID:EPI_ISL-499083) was provided by InViV group, UNC, Argentina. Omicron BA.5 variant (GISAID ID: EPI_ISL-16297058) was provided by IIB group, UNSAM, Argentina.

### 2.5 Quantitation of plasma SARS-CoV-2–specific IgG antibodies

IgG antibodies to the SARS-CoV-2 spike protein were detected using an ELISA kit (COVIDAR, Lemos lab). Anti-spike IgG antibody titers were determined by endpoint titration, defined as the reciprocal of the highest dilution of serum that gives a reading above the cut-off (Ojeda et al., 2021).

### 2.6 Neutralization assay

Neutralization assays were performed as we previously described (Seery et al., 2022). Briefly, deidentified plasma samples were heat-inactivated at 56°C for 20 min. Two-fold serial dilutions were incubated at 37°C for 1 h with Wuhan and Omicron BA.5 variants (MOI=0.01) in DMEM with 2% FBS. Fifty µL of the mixtures were added to Vero E6 cell monolayers for 1 h at 37°C in 96-well plates. After removing the infectious media, DMEM with 2% FBS was added. Cells were cultured for 72 h, fixed with 4% paraformaldehyde, and stained with crystal violet. Inhibitory concentrations of 50% (IC50) values were calculated.

### 2.7 Expression of activation-induced markers in T cells

The activation of antigen-specific T cells was assessed by measuring the percentage of AIM+ cells: (OX40^+^CD137^+^) for CD4^+^ T cells and (CD69^+^CD137^+^) for CD8^+^ T cells. Peripheral blood mononuclear cells (PBMCs) were stimulated with overlapping peptide megapools corresponding to the Wuhan and/or Omicron BA.5 sequences, provided by the Sette Lab (La Jolla Institute of Immunology, CA, USA) (Grifoni et al., 2020; Dan et al., 2021; Grifoni et al., 2021). Thawed PBMCs were rested for 2 hours at 37°C in RPMI 1640 medium (Sigma-Aldrich) supplemented with 10% heat-inactivated human AB serum, 2 mM L-glutamine, and penicillin-streptomycin (all from Sigma-Aldrich). A total of 1×10^6^ cells per well were seeded in U-bottom 96-well plates and stimulated with 1 µg/mL of SARS-CoV-2 peptide megapools for 24 hours. Phytohemagglutinin (PHA, 5 µg/mL; Sigma-Aldrich) were used as a positive control, and an equimolar amount of DMSO (Sigma-Aldrich) as a negative control. Supernatants were collected at 24 hours post-stimulation for IFN-γ detection. Cells were washed and stained with Zombie NIR Live/Dead Stain and the following antibody panel: CD19 PE, CD4 BV605, CD8 FITC, CD137 BV421, CD69 AF647, and OX40 PerCP-Cy5.5 (BioLegend). Data were acquired using a Northern Lights flow cytometer and analyzed with SpectroFlo software (Cytek). T-cell data were calculated either as background-subtracted values or as a stimulation index. Background-subtracted values were obtained by subtracting the percentage of AIM+ cells following DMSO stimulation from those observed after peptide-stimulation. Stimulation index was calculated as the ratio between the percentage of AIM+ cells after stimulation with SARS-CoV-2 peptides and the percentage of AIM+ cells cultures without stimulating peptides. An index >2 fold change was considered positive.

### 2.8 Measurement of IFN-γ by ELISA

It was performed in cell supernatants following manufacturer´s instructions (BD Biosciences).

### 2.9 Statistics

Clinical characteristics were summarized using descriptive statistics. Categorical variables are reported as numbers and percentages. Quantitative variables are reported as medians and interquartile ranges and presented as medians and minimum to maximum in the figures. Two groups were compared using the Mann-Whitney U test. Two groups’ proportions were compared using the Chi-square test and Fisher exact test. Data were calculated using GraphPad Prism V.9. A p-value <0.05 was considered statistically significant.

## 3 Results

### 3.1 Vaccinated immunocompromised children exhibit lower levels of neutralizing antibodies against SARS-CoV-2 compared with healthy children

**Figure 1:**
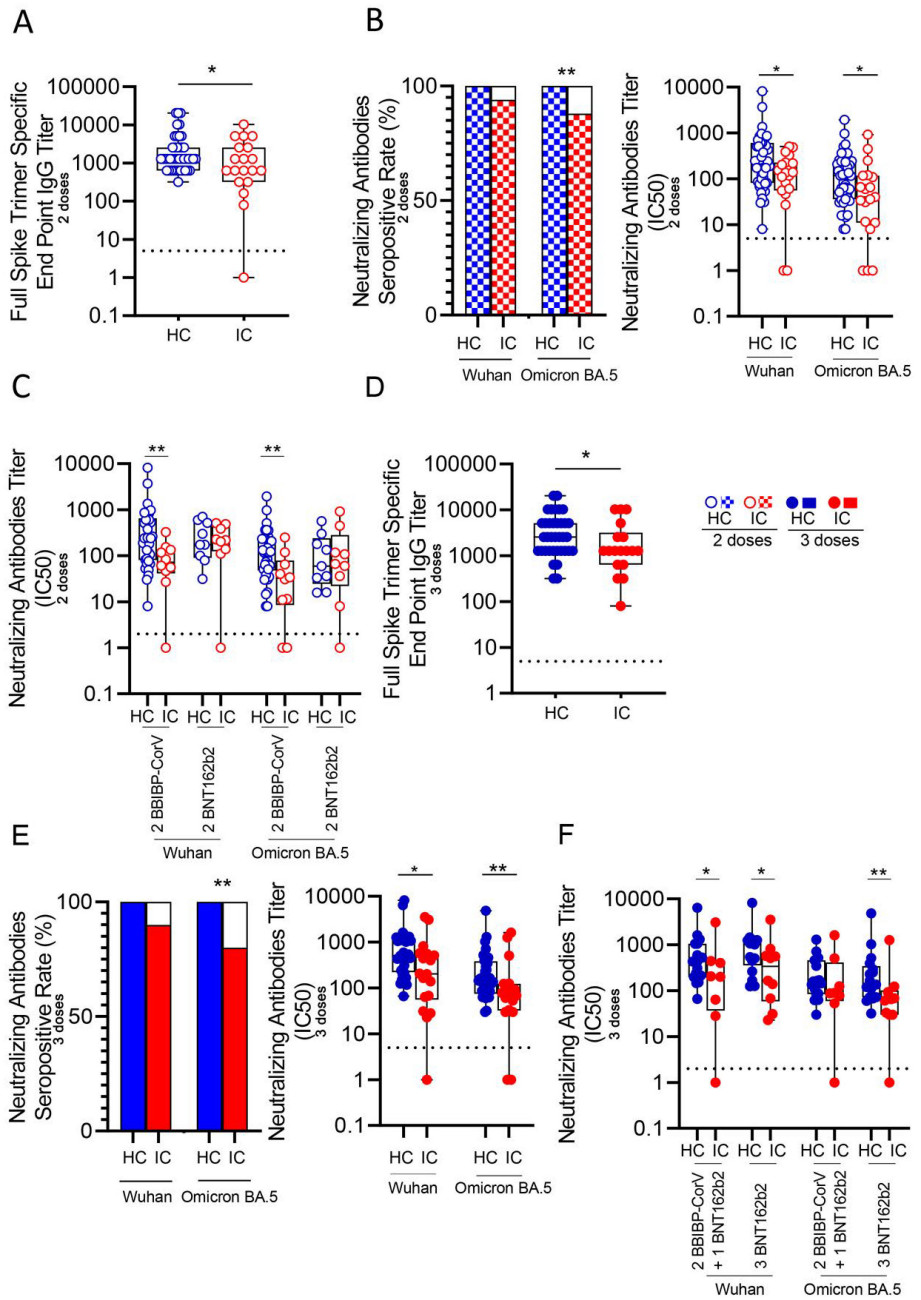
Antibody response against Wuhan and Omicron BA.5 variants in vaccinated IC and HC. **(A)** Titers of IgG anti-spike antibodies defined by end point dilution in plasma from IC (n=19) and HC (n=43) receiving two-doses of COVID-19 vaccines. **(B)** Neutralizing activity against Wuhan and Omicron BA.5 variants in plasma from IC and HC receiving two-doses of COVID-19 vaccines. Left: Bars show the percentage of positive samples for neutralizing activity. Right: Neutralization antibody titers determined by the reciprocal IC50. **(C)** Neutralizing activity against Wuhan and Omicron BA.5 variants in plasma from children receiving two-doses of BBIBP-CorV (IC, n=10 and HC, n=34) or BNT162b2 (IC, n=9 and HC, n=9) vaccines. **(D)** Titers of IgG anti-spike antibodies defined by end point dilution in plasma from IC (n=18) and HC (n=36) receiving three-doses of COVID-19 vaccines. **(E)** Neutralizing activity against Wuhan and Omicron BA.5 variants in plasma from IC and HC receiving three-doses of COVID-19 vaccines. Left: Bars show the percentage of positive samples for neutralizing activity. Right: Neutralization antibody titers determined by the reciprocal IC50. **(F)** Neutralizing activity against Wuhan and Omicron BA.5 variants in plasma from children receiving three-doses of COVID-19 vaccines: 2 doses BBIBP-CorV plus 1 dose BNT162b2 (IC, n=8 and HC, n=19) or 3 doses of BNT162b2 (IC, n=10 and HC, n=17). Dotted line indicates the limit of detection value. Median and min to max of n donors are shown in **(A, B)** right, **(C–E)** right and **(F)** P values were determined by Pearson’s Chi square test (B left and E left) and Mann-Whitney U test **(A, B)** right, **(C–E)** right and **(F)**. *p<0.05, **p<0.01. HC (blue circle), IC (red circle), two-doses (open circle), three-doses (filled circle), negative (white square), positive two-doses (dotted square), positive three-doses (filled square).

We analyzed serum antibody levels against SARS-CoV-2 in IC and HC following vaccination with two doses of either BBIBP-CorV or BNT162b2 vaccines. Blood samples were collected 13 to 17 months post-vaccination. Detailed participant information is provided in **Table 1**; **Supplementary Table 1**. Titers of anti-SARS-CoV-2 IgG and neutralizing antibodies across the study cohorts are shown in **Supplementary Table 2**. Four children receiving EV gammaglobulin were excluded from this analysis. Results showed that IC (n=19) had significantly lower IgG titers against the SARS-CoV-2 spike protein compared to HC (n=43, p<0.05; **Figure 1A**). Most children in both groups exhibited neutralizing antibodies against both, Wuhan and Omicron BA.5 variants long time after vaccination. However, IC demonstrated lower seropositivity for the Omicron BA.5 variant (84%, 16/19) compared to HC (100%, 43/43; p<0.01, **Figure 1B** left). Neutralizing antibody titers against SARS-CoV-2 were significantly reduced in IC for both variants (p<0.05, **Figure 1B** right). When comparing vaccine types, IC receiving BBIBP-CorV had lower neutralizing antibody levels for both variants than HC (p<0.01 for both Wuhan and Omicron BA.5). No significant differences were found between IC and HC who received the BNT162b2 vaccine (**Figure 1C**).

The lower antibody response in IC was also observed after three vaccine doses. Four children receiving EV gammaglobulin were excluded from this analysis. IC (n=18) had lower anti-SARS-CoV-2 IgG titers compared to HC (n=36, p<0.05; **Figure 1D**). Seropositivity for Omicron BA.5 was lower in IC (83%, 15/18) compared to HC (100%, 40/40; p<0.01, **Figure 1E** left). Neutralizing titers in IC were significantly decreased for both variants (p<0.05 for Wuhan and p<0.01 for Omicron BA.5; **Figure 1E** right). When analyzing vaccine regimens, a lower response in IC was observed overall (p<0.05), except for neutralizing antibodies against Omicron BA.5 in those receiving two doses of BBIBP-CorV followed by a third dose of BNT162b2, where no differences were found between IC and HC (**Figure 1F**). When grouping immunocompromised children based on their underlying conditions, among those vaccinated with two doses, 8 had cancer, 4 had undergone transplantation, 2 had autoimmunity and 9 had IIE. In the cohort receiving three doses, 7 had cancer, 5 had autoimmune diseases, 5 had undergone transplantation, and 5 had IIE. For the analysis, only subgroups with a minimum of three children were included, and those children receiving EV gammaglobulin were excluded. As shown in **Supplementary Figure S1**, we observed a lower neutralizing antibody response in IC compared to HC across all subgroups. However, statistically significant differences were only found between HC and the transplanted subgroup (p<0.05 for Wuhan and Omicron BA.5; p<0.01 for Omicron BA.5 in the two- and three-dose groups, respectively). Finally, as expected, we found a significant reduction in neutralizing titers against the Omicron variant compared to the Wuhan variant by analyzing paired samples in both HC (p< 0.0001 for Wuhan vs. Omicron BA.5 after 2 and 3 doses) and IC groups (p< 0.01 and p< 0.001 for Wuhan vs. Omicron BA.5 after 2 and 3 doses, respectively; **Supplementary Figures S2A, B**).

### 3.2 Vaccinated immunocompromised children display a decreased CD8+T cell memory response compared with healthy children

**Figure 2:**
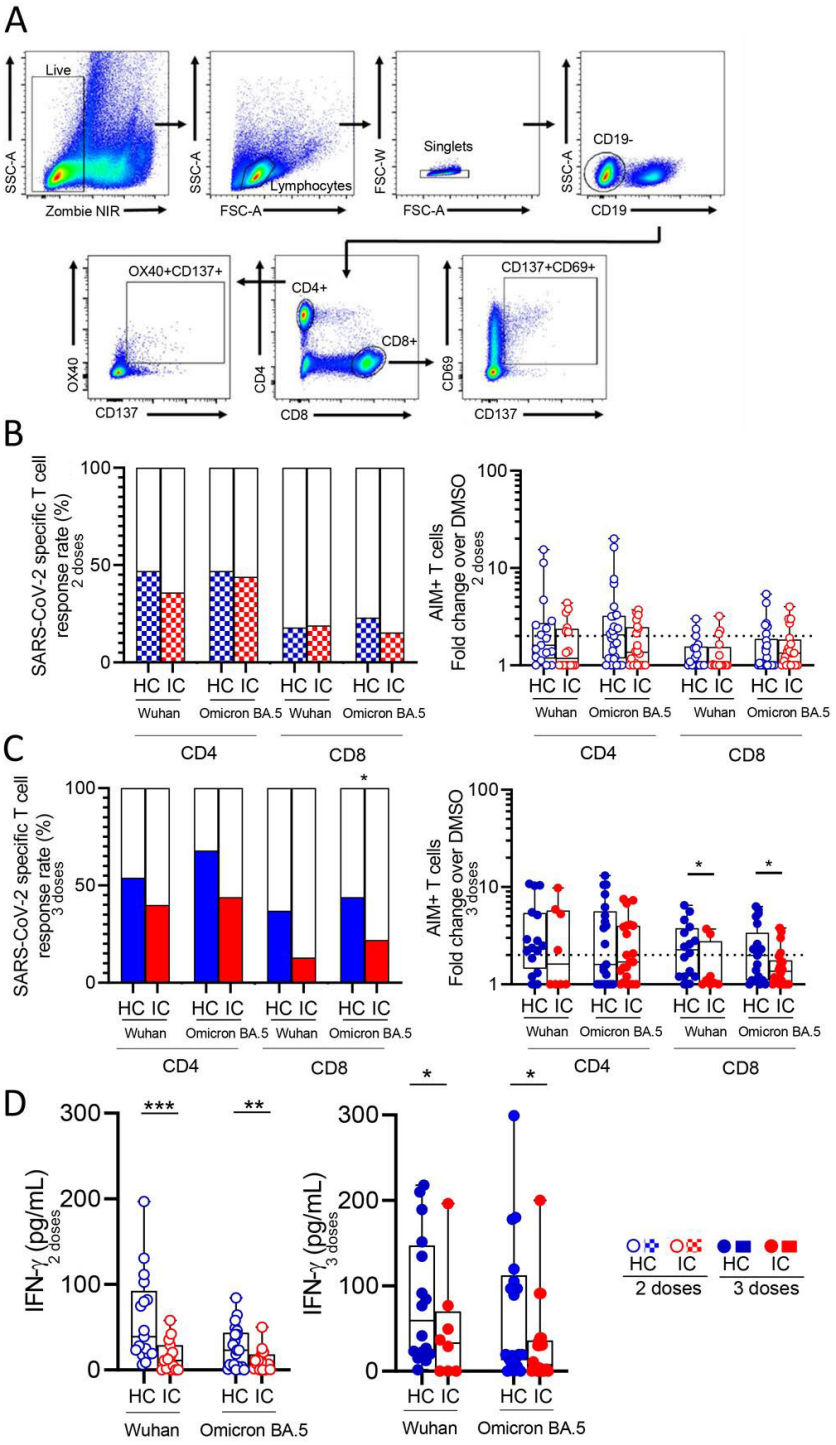
SARS-CoV-2 specific T cell response in vaccinated IC and HC. Antigen-specific T cells were measured as a percentage of CD4+OX40+CD137+ and CD8+CD69+CD137+ T cells after stimulation of PBMCs from children receiving two or three doses of COVID-19 vaccines with CD4_S and CD8_S peptide megapools of Wuhan and Omicron BA.5 compared to negative control (DMSO) analyzed by flow cytometry. **(A)** Gating strategies to define SARS-CoV-2-specific CD4+ and CD8+ T cells by flow cytometry. **(B)** Left: Bars show the percentage of IC and HC vaccinated with 2 doses that presented circulating specific CD4+ and CD8+ T cell response against Wuhan (IC, n=16 and HC, n=17) and Omicron BA.5 (IC, n=20 and HC, n=26). Right: Stimulation index quantitation of AIM+ T cells (fold change over the DMSO condition). **(C)** Left: Bars show the percentage of IC and HC vaccinated with 3 doses that presented circulating specific CD4+ and CD8+ T cell response against Wuhan (IC, n=8 and HC, n=16) and Omicron BA.5 (IC, n=22 and HC, n=21). Right: Stimulation index quantitation of AIM+ T cells (fold change over the DMSO condition). **(D)** Levels of IFN-γ in the supernatant culture of PBMCs from IC and HC stimulated with peptide megapools of Wuhan and Omicron BA.5 determined by ELISA. Dotted line indicates the fold change ≥ 2. Median and min to max of n donors are shown in **(B)** right, **(C)** right and **(D)** P values were determined by Pearson’s Chi square test **(B)** left and **(C)** left] and Mann-Whitney U test **(B)** right, **(C)** right and **(D)**]. *p<0.05, **p<0.01, ***p<0.01. HC (blue circle), IC (red circle), two-doses (open circle), three-doses (filled circle), negative (white square), positive two-doses (dotted square), positive three-doses (filled square).

The memory response to SARS-CoV-2, mediated by either CD4+ and CD8+ T cells, was analyzed by flow cytometry following overnight stimulation of PBMCs with peptide megapools derived from the Wuhan and Omicron BA.5 variants. These peptide pools specifically stimulated CD4+ and CD8+ T cells. The gating strategy used for analysis is illustrated in **Figure 2A**. Approximately 50% of IC and HC showed a significant CD4+ T cell response to both variants after receiving two vaccine doses, while less than 20% displayed a significant CD8+ T cell response (IC, n=16 and n=20, HC, n=17 and n=26 for Wuhan and Omicron BA.5 response respectively; **Figure 2B** left). To quantify this response, we calculated the fold change in CD4+ and CD8+ T cell activity as the ratio of positive T cells for stimulated and unstimulated cells for each donor. No significant differences in this stimulation index were found between the HC and IC groups for either CD4+ or CD8+ T cells (**Figure 2B** right). A similar analysis was performed in children who received three vaccine doses. Over 40% of children in both cohorts showed a positive CD4+ T cell response to Wuhan and Omicron BA.5 variants, with no significant differences between the cohorts. For CD8+ T cells, IC had a lower response than HC, but a significant difference (p<0.05) was only observed in response to Omicron BA.5-derived peptides (IC, n=8 and n=22, HC, n=16 and n=21 for Wuhan and Omicron BA.5 respectively; **Figure 2C** left). Consistent with these findings, the CD4+ T cell stimulation index was similar across cohorts, while CD8+ T cells in IC showed a lower response to Wuhan and Omicron BA.5 peptides (p<0.05; **Figure 2C** right). Similar conclusions were obtained using DMSO-subtracted values as metrics for both specific CD4+ and CD8+ T cell response following two or three vaccine doses (**Supplementary Figure S3**).

Moreover, we observed a comparable T cell memory response against both the Wuhan and Omicron BA.5 variants in both HC and IC (**Supplementary Figures S2C, D**). We finally analyzed IFN-γ levels in supernatants of PBMCs from children who received two or three vaccine doses, stimulated with Wuhan and BA.5 peptides for 24 hours. In all cases, IFN-γ levels were lower in PBMCs from IC compared to HC (p<0.001 and p<0.01 for two doses against Wuhan and Omicron BA.5; p<0.05 for three doses against both variants; **Figure 2D**).

### 3.3 Integrated analysis of memory B and T cell responses

It was valuable to explore different relationships between the B and T memory compartments and analyze combinations of positive or negative responses by reexamining the data of neutralizing antibodies against the Wuhan and/or Omicron BA.5 variants (“N”), as well as antigen-specific CD4^+^ (“4”) and CD8^+^ (“8”) T cell responses targeting the Spike protein variants. This analysis was conducted exclusively in children who received three doses of the vaccine, and only in those children for whom both antibody memory and T cell memory could be assessed.

**Figure 3:**
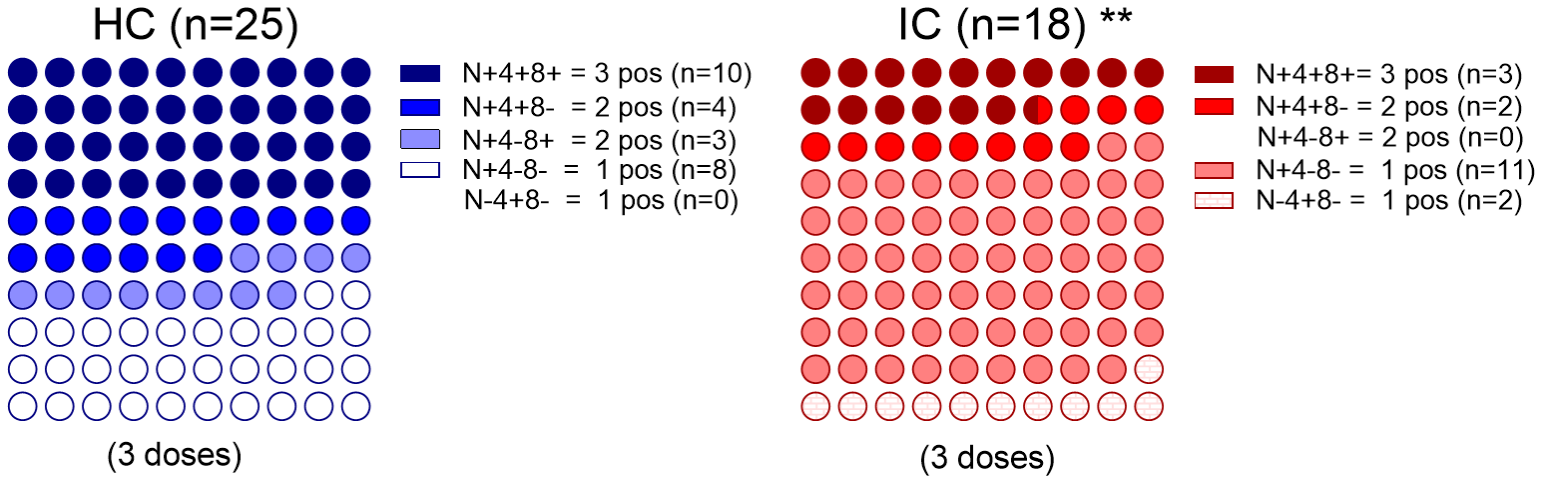
Immune memory components relationship. Percentage dot plots showing frequencies (normalized to 100%) of IC (n=18) and HC (n=25) participants who received 3 doses with indicated immune memory components evaluated. Comparison of positive responses in more than one of the parameters analyzed (antibodies, CD4+ T cell response and CD8+ T cell response) is shown. **p<0.01. Pearson’s Chi square test. HC has no cases in the category N-4+8- and IC has no cases in the category N+4-8+. N, neutralizing antibodies, 4, SARS-CoV-2–specific CD4+ T cells; 8, SARS-CoV-2–specific CD8+ T cells. P values were determined by Pearson’s Chi square test, **p<0.01.

In the HC group (n=25), all children exhibited an immune response: 40% were positive across all three immune compartments, 28% had neutralizing antibodies along with either a CD4+ or CD8+ T cell response, and the remaining 32% showed only neutralizing antibodies. Although all IC (n=18) children also exhibited some kind of immune response, the pattern was different. While most of children had only neutralizing antibodies (61%), 17% of them were positive across all three immune compartments, 11% had neutralizing antibodies along with CD4+ T cell response, and the remaining 11% had only positive CD4+ T cell response. Comparison of children with positive responses in more than one of the parameters analyzed (antibodies, CD4+ T cell response and CD8+ T cell response) revealed that the IC group had a more restricted response than the HC group (p < 0.01, **Figure 3**).

## 4 Discussion

Vaccination has played a crucial role in addressing the pandemic (Watson et al., 2022), with memory B and T cell responses being key contributors to long-term protection (Sette and Crotty, 2021). During the Omicron period, two or more doses of the original monovalent COVID-19 vaccine prevented hospitalizations in children aged 5–18 (Price et al., 2022). However, optimal immunity requires a fully functional immune system, which IC often lack. Treatments such as B-cell depletion therapies, high-dose glucocorticoids, tacrolimus and mycophenolate mofetil among others, weaken the response to vaccines (Deepak et al., 2021; Friedman et al., 2021; Lee et al., 2022). This highlights the importance of booster doses, especially for immunocompromised individuals. However, the administration of boosters has significantly decreased both in Argentina and worldwide (Lazarus et al., 2023), and this decrease includes IC patients (Zheng et al., 2023). It’s concerning that few studies have examined how the memory immune response against SARS-CoV-2 lasts over time in IC.

For instance, children with pediatric inflammatory bowel disease showed an antibody response to monovalent mRNA vaccines similar to HC. However, those on anti-TNF-α therapy, but not Infliximab or Adalimumab, had a weaker response (Cotugno et al., 2022). A study lacking a HC cohort found that most children with solid organ transplants, stem cell transplants, and rheumatologic diseases had detectable antibody and T cell responses after the second dose of mRNA vaccines, which improved after the third dose. Notably, while all children showed a CD4+ T cell response after the third dose, only 44% had a CD8+ T cell response (Morgans et al., 2023). Additionally, marked differences in immune responses were observed between liver transplant and IgA nephropathy patients following a standard two-dose mRNA regimen being these differences only partially explained by the different immunosuppressive treatments used (Lu et al., 2023). Two additional studies also reported substantial differences in the immune response induced by mRNA vaccines in IC according the underlying condition or treatment, being kidney transplant recipients who showed a significant reduction in both, the humoral and cellular response (Haskin et al., 2021; Lalia et al., 2023). Furthermore, a recent study found that while children with different immunocompromising conditions developed immune responses comparable to healthy children, solid organ transplant recipients had lower levels of neutralizing antibodies and reduced frequencies of Tregs and Bregs six months post-mRNA vaccination (Di Chiara et al., 2024). Data on inactivated COVID-19 vaccines in immunocompromised children are limited. One study reported that administering inactivated vaccines followed by an Ad5-nCoV booster in pediatric liver transplant recipients elicited a strong humoral response but a weak T-cell response (Zheng et al., 2024).

While previous studies primarily examined the short-term B and T cell responses to SARS-CoV-2 vaccination, we focused on the durability of the memory response 13 to 17 months post-vaccination. In our cohorts, we observed that IC who received two vaccine doses of BBIBP-CorV showed a lower neutralizing antibody response against both Wuhan and Omicron BA.5 variants, compare with HC. The lower immunogenicity of the inactivated BBIBP-CorV vaccine may explain this observation. Even with three doses, IC had lower neutralizing antibody levels against both variants compared to HC. As expected, we found that the titers of neutralizing antibodies against Omicron were lower than those directed against the Wuhan variant, both in HC and IC.

When examining the T cell response, our findings revealed three key observations. First, fewer than 25% of children in both cohorts exhibited a specific CD8+ T cell response after two doses, with IC showing a more pronounced reduction after three doses compared to HC. Second, and consistent with previous observations (Tarke et al., 2022),we observed that first-generation COVID-19 vaccines induced a similar T-cell response against both, Omicron and Wuhan variants, in both cohorts. Third, IC showed a decreased production of IFN-γ by stimulated PBMCs, reinforcing the notion of a suboptimal T cell memory response. This is consistent with Rosnik et al, which demonstrated a skewed T cell response in IC patients, favoring IL-2 and TNF-α production over IFN-γ, even after receiving updated vaccines (Roznik et al., 2024). Finally, by analyzing the two compartments of the adaptive immune response in an integrated manner, we observed that while there is a decreased proportion of immune memory components in the IC group, our findings also underscore the heterogeneity of immune memory, revealing distinct patterns among different individuals.

This study has several limitations. The small sample size of the IC cohort means that our observations, particularly those related to the stratification of patients into different subgroups based on their underlying pathology and/or treatment regimens, should be validated in a study with a larger cohort. We were unable to conduct a longitudinal analysis of the immune response over time in our cohorts, as we did not have successive samples from each child at baseline and after the second and third vaccine doses. We also could not perform assays to analyze T cell responses using seasonal coronavirus antigens as control stimuli. Furthermore, all participants received monovalent vaccines rather than the bivalent vaccines targeting the predominant Omicron variants.

Given the ongoing threat of COVID-19 and other respiratory viruses, updated vaccines and boosters are critical to preventing severe disease, especially for children with immunocompromising conditions as well as in developing regions where booster coverage remains low and unevenly distributed. Ensuring access to boosters in these areas is essential to mitigating health disparities and controlling viral spread post-pandemic.

## Data Availability

The raw data supporting the conclusions of this article will be made available by the authors, without undue reservation.
