# Supplementary material for: Immunological memory to COVID-19 vaccines in immunocompromised and immunocompetent children
Source: Front Cell Infect Microbiol. 2025 Feb 17;15:1527573. doi: 10.3389/fcimb.2025.1527573 (PMC11873107; doi:10.3389/fcimb.2025.1527573)
Supplement: Supplementary file 1 [file Presentation1.pdf]

Supplementary Table 1

Patient	Vaccine Doses	Age	Gender	Medical history	Treatment
1	2	14	Female	Stem cell transplant	Tacrolimus-Prednisone
2	2	13	Male	SLE	Azatioprine-Prednisone-Hydroxychloroquine
3	2	11	Male	Bone tumor	Chemotheraphy-Radiotheraphy
4	2	8	Male	IgA deficiency	
5	2	10	Female	Hypogammaglobulinemia	Gammaglobuline
6	2	5	Male	XLPD Type 1	Gammaglobuline
7	2	5	Female	SCID	Gammaglobuline
8	2	10	Female	SLE	Methylprednisolone-Prednisone
9	2	12	Male	Soft tissue tumour	Chemotheraphy
10	2	9	Female	ALL	Chemotheraphy
11	2	15	Female	IIE	
12	2	13	Male	CGD	
13	2	11	Female	Chronic mucocutaneous candidiasis	
14	2	7	Male	XLA	Gammaglobuline
15	2	10	Male	IgA deficiency	Prednisone-Mezalazine.
16	2	10	Male	Stem cell transplant	Prednisone-Tacrolimus
17	2	10	Female	AML	Chemotheraphy
18	2	17	Male	AML	Chemotheraphy
19	2	13	Female	ALL	Chemotheraphy
20	2	13	Female	ALL	Corticosteroids- Vincristine-Daunorubicin
21	2	12	Male	ALL	Chemotheraphy
22	2	11	Male	Stem cell transplant	Tacrolimus-Prednisone
23	2	7	Male	Kidney transplant	Tacrolimus-Prednisone-Mycophenolate mofetil
24	3	13	Male	CVID	Gammaglobuline
25	3	14	Male	Stem cell transplant	Chemotheraphy-Radiotheraphy
26	3	9	Female	Kidney transplant	Tacrolimus-Prednisone-Mycophenolate mofetil
27	3	8	Male	IgA deficiency	
28	3	12	Female	Stem cell transplant	Tacrolimus-Prednisone
29	3	7	Male	XLA	Gammaglobuline
30	3	8	Male	ALL	Corticosteroids- Vincristine-Daunorubicin
31	3	12	Female	IIE- Stem cell transplant	Gammaglobuline- Tacrolimus-Prednisone
32	3	15	Male	ALL	Corticosteroids
33	3	15	Male	ALL	Corticosteroids
34	3	14	Male	SLE	Cyclophosphamide
35	3	10	Male	CVID	Gammaglobuline
36	3	9	Male	ALL	Corticosteroids- Vincristine-Daunorubicin
37	3	16	Female	AML	Chemotheraphy
38	3	13	Male	ALL	Corticosteroids- Vincristine-Daunorubicin
39	3	8	Male	Renal insufficiency	Corticosteroids
40	3	6	Male	Kidney tumour	Chemotheraphy
41	3	14	Male	Reumatoid arthritis	Corticosteroids
42	3	16	Male	SLE	Cyclophosphamide- Corticosteroids

43	3	14	Male	Kidney transplant	Tacrolimus-Prednisone-Mycophenolate mofetil
44	3	6	Female	IIE	
45	3	14	Male	Fibromyalgia	Corticosteroids

Abbreviations: SLE, Systemic Lupus Erythematosus; XLPD Type 1, X-Linked Lymphoproliferative Disease Type; SCID, Severe Combined Immunedeficiency; ALL, Acute Lymphoblastic Leukemia; IIE, Innate Inborn Error; CGD, Chronic Granulomatous Disease; XLA, X-linked agammaglobulinemia; AML, Acute Myeloid Leukemia; CVID, Common variable immunodeficiency.

Supplementary Table 2. Titers of anti-SARS-CoV-2 IgG and neutralizing antibodies across the study cohorts

	Children		
	Healthy	Immunocompromised	
Full Spike Trimer specific IgG titer			
2 doses	1280 (800-2560), n=43	640 (320-2560), n=19	*
3 doses	2560 (1280-5120), n=36	1280 (640-3200), n=18	*
Neutralizing ab titer (Wuhan)			
2 doses	245 (83-610), n=43	135 (56-227), n=19	*
3 doses	505 (176-1212), n=36	204 (56-563), n=18	*
Neutralizing ab titer (Omicron BA.5)			
2 doses	123 (41-243), n=43	40 (11-118), n=19	*
3 doses	140 (75-381), n=36	73 (31-123), n=18	**
Full spike Trimer specific IgG titer			
2 doses (BBIBP-CorV)	1280 (640-2560), n=34	640 (280-1600), n=10	*
2 doses (BNT162b2)	1280 (1280-3840), n= 9	1280 (480-5120), n=9	
3 doses (2 BBIBP-CorV+ 1 BNT162b2)	2560 (1280-5120), n=19	1208 (560-2240), n=8	
3 doses (BNT162b2)	5120 (1280-10240), n=17	1280 (640-10240), n=10	
Neutralizing ab titer (Wuhan)			
2 doses (BBIBP-CorV)	243 (81-654), n=34	72 (42-140), n=10	**
2 doses (BNT162b2)	175 (90-558), n=9	219 (125-438), n=9	
3 doses (2 BBIBP-CorV+ 1 BNT162b2)	434 (145-1053), n=19	204 (37-434), n=8	*
3 doses (BNT162b2)	1022 (356-1249), n=17	340 (59-631), n=10	*
Neutralizing ab titer (Omicron BA.5)			
2 doses (BBIBP-CorV)	120 (48-241), n=34	33 (8-79), n=10	**
2 doses (BNT162b2)	60 (25-240), n=9	66 (22-283), n=9	
3 doses (2 BBIBP-CorV+ 1 BNT162b2)	144 (84-459), n=19	89 (59-414), n=8	
3 doses (BNT162b2)	135 (72-343), n=17	49 (30-101), n=10	**

Eight children receiving IVIG were excluded from the analysis. Median (IQR) of titers are shown. *$p<0.05$, **$p<0.01$.

Supplementary Figure 1.

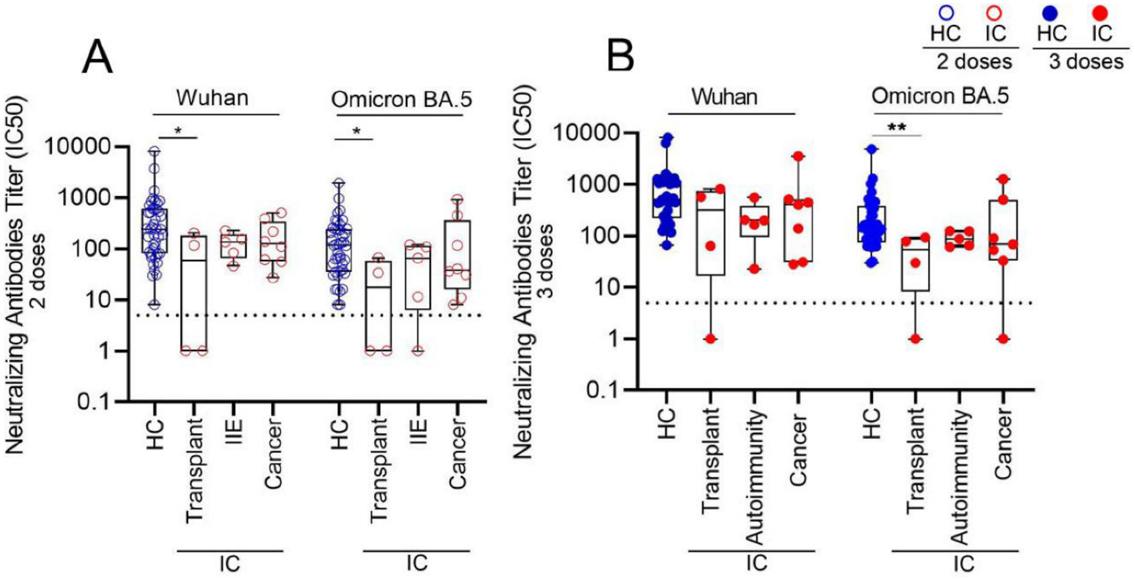

Figure S1. Antibody response against Wuhan and Omicron BA.5 variants in vaccinated HC and IC divided according to their underlying disease. (A) Neutralizing activity against Wuhan and Omicron BA.5 variants in plasma from HC (n=43), children undergoing transplantation (n=4), children with IIE (n=5) and children with cancer history (n=8) receiving two-doses of COVID-19 vaccines. Four children receiving EV gammaglobulin were excluded from this analysis. **(B)** Neutralizing activity against Wuhan and Omicron BA.5 variants in plasma from HC (n=36), children undergoing transplantation (n=4), children with autoimmune disease (n=5) and children with cancer history (n=7) receiving two-doses of COVID-19 vaccines. Four children receiving EV gammaglobulin were excluded from this analysis. Neutralization antibody titers were determined by the reciprocal IC50. Dotted line indicates the limit of detection value. Median and min to max of n donors are shown. Mann-Whitney U test. * $p<0.05$, ** $p<0.01$. HC (blue circle), IC (red circle), two-doses (open circle), three-doses (filled circle).

Supplementary Figure 2.

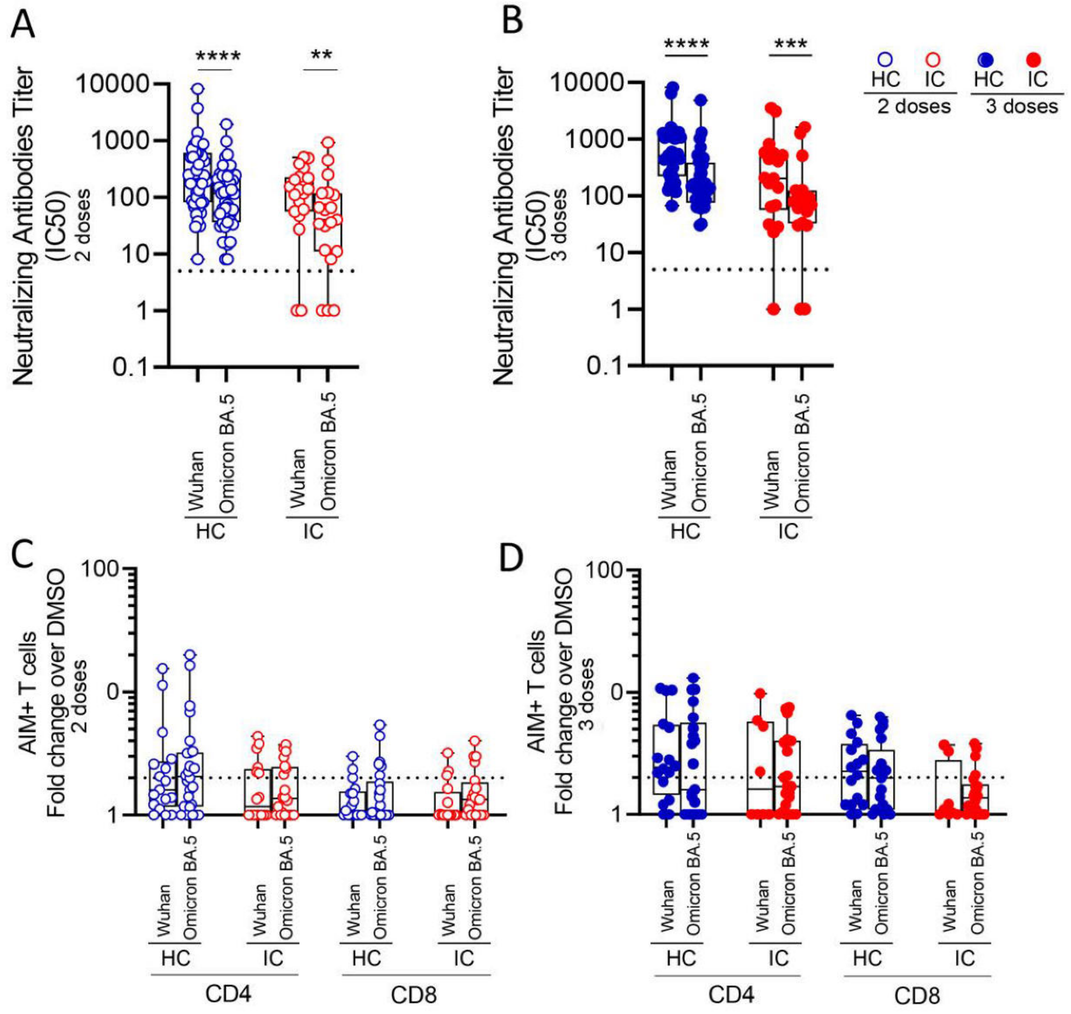

Figure S2. Cross-reactivity against Wuhan and Omicron BA.5 variants regarding neutralizing antibodies and specific T cells in HC and IC. (A-B) Comparison of neutralization antibody titres against the Wuhan and Omicron BA.5 variants in paired samples from children receiving two-doses (A, HC n=43 and IC n=19) and three-doses (B, HC n=36 and IC n=18). Neutralization antibody titers were determined by the reciprocal IC50. Children receiving EV gammaglobulin were excluded from this analysis. Dotted line indicates the limit of detection value. **(C-D)** Comparison of antigen-specific T cells measured as a percentage of CD4+OX40+CD137+ and CD8+CD69+CD137+ T cells after stimulation of PBMCs from children receiving two (C, HC n=17 and IC n=16 for Wuhan; HC n=26 and IC n=20 for Omicron BA.5) or three doses (D, HC n=16 and IC n=8 for Wuhan; HC n=21 and IC n=22 for Omicron BA.5) of COVID-19 vaccines with CD4_S and CD8_S peptide megapools of Wuhan and Omicron BA.5 compared to negative control (DMSO) analyzed by flow cytometry. Fold change over the DMSO condition is shown. Dotted line indicates the fold change ≥ 2. Median and min to max of n donors are shown. Mann-Whitney U test. ** p<0.01, ***p<0.001, ****p<0.0001. HC (blue circle), IC (red circle), two-doses (open circle), three-doses (filled circle).

Supplementary Figure 3.

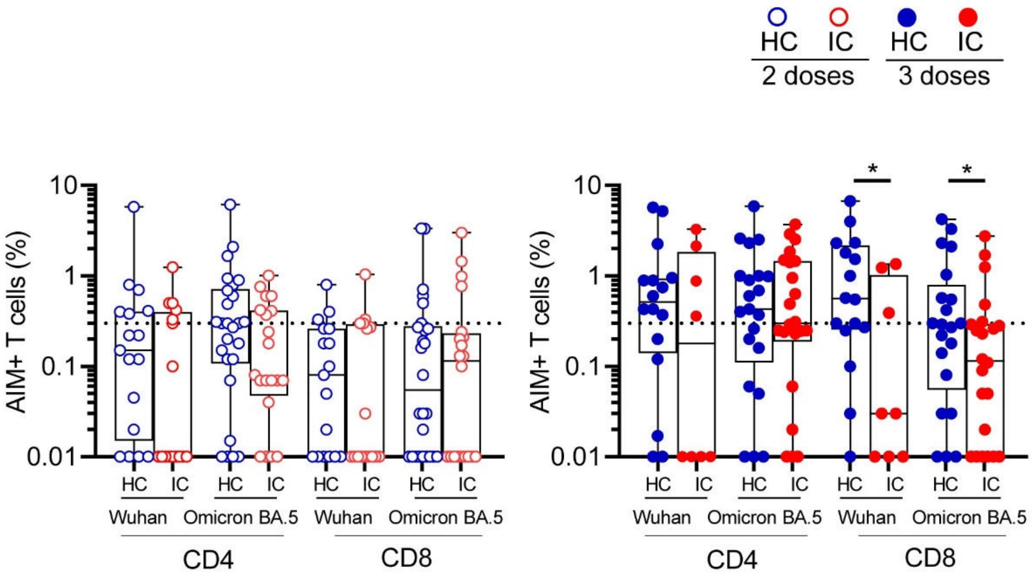

Figure S3. SARS-CoV-2 specific T cell response against Wuhan and Omicron BA.5 variants. Antigen-specific T cells were measured as a percentage of CD4+OX40+CD137+ and CD8+CD69+CD137+ T cells after stimulation of PBMCs with CD4_S and CD8_S peptide megapools of Wuhan and Omicron BA.5, analyzed by flow cytometry. Data were background subtracted against DMSO negative control. **(A)** Percentage of AIM+ T cells in IC and HC following vaccination with 2 doses against Wuhan (IC, n=16 and HC, n=17) and Omicron BA.5 (IC, n=20 and HC, n=26). **(B)** Percentage of AIM+ T cells in IC and HC following vaccination with 3 doses against Wuhan (IC, n=8 and HC, n=16) and Omicron BA.5 (IC, n=22 and HC, n=21). Median and min to max of n donors are shown. Mann-Whitney U test. * $p<0.05$. HC (blue circle), IC (red circle), two-doses (open circle), three-doses (filled circle).
